# Supplementary material for: Impact of dynamic electrostatic disorder on hole mobility in rubrene: a nonadiabatic molecular dynamics investigation
Source: J Mater Chem C Mater. 2026 Sep 23. Online ahead of print. doi: 10.1039/d6tc01991a (PMC13628186; doi:10.1039/d6tc01991a)
Supplement: Supplementary file 1 [file TC-OLF-D6TC01991A-s001.pdf]

Supplementary Information (SI) for Journal of Materials Chemistry C.
This journal is © The Royal Society of Chemistry 2026

Supplementary Information

Impact of dynamic electrostatic disorder on hole mobility in rubrene: a non-adiabatic molecular dynamics investigation

Jan Elsner,[a,b,c] Samuele Giannini,[d] and Jochen Blumberger*[a]

[a] *Department of Physics and Astronomy and Thomas Young Centre, University College London, London WC1E 6BT, U.K.*

[b] *Lehrstuhl für Theoretische Chemie II, Ruhr-Universität Bochum, 44780 Bochum, Germany.*

[c] *Research Center Chemical Sciences and Sustainability, Research Alliance Ruhr, 44780 Bochum, Germany.*

[d] *Department of Chemistry and Industrial Chemistry, University of Pisa, Pisa, Italy.*

* *Email: j.blumberger@ucl.ac.uk*

S1 Reorganization energy for individual dimers

Table 1 of the main text (Section 2.1) reports reorganization energies for hole transfer for P- and T-type dimers, where in each case results are averaged over two symmetry-related dimers, shown in Fig. 1 of the main text. Results for the individual dimers $P1$, $P2$, $T1$, and $T2$, as well as the corresponding averaged values for P and T, are listed in Table S1.

Table S1 Computed reorganization energies for hole transfer along individual dimers $P1$, $P2$, $T1$, and $T2$ in rubrene using different treatments of electrostatic interactions. The direction-averaged values P and T are also shown.

Method	Dimer[a]	λ^{st} / meV[b]	λ^{var} / meV[c]
Zero charge	$P1$	152	156 ± 3
Zero charge	$P2$	152	154 ± 3
Zero charge	P	152	155 ± 2
Zero charge	$T1$	152	160 ± 4
Zero charge	$T2$	152	151 ± 3
Zero charge	T	152	155 ± 5
DSF	$P1$	175	176 ± 3
DSF	$P2$	175	172 ± 4
DSF	P	175	174 ± 3
DSF	$T1$	182	190 ± 4
DSF	$T2$	182	185 ± 4
DSF	T	182	187 ± 4
SPME	$P1$	181	190 ± 4
SPME	$P2$	181	189 ± 4
SPME	P	181	190 ± 3
SPME	$T1$	191	197 ± 4
SPME	$T2$	191	196 ± 4
SPME	T	191	197 ± 3

[a] Dimer considered for hole transfer (see Fig. 1 of the main text). P and T denote the averages over the two symmetry-related dimers, as reported in Table 1 of the main text.
[b] Stokes shift reorganization energy, λ^{st}, Eq. 5 of the main text. All statistical uncertainties for λ^{st} are smaller than 1 meV.
[c] Fluctuation-derived reorganization energy, λ^{var}, Eq. 6 of the main text, and statistical uncertainty. For individual dimer rows, uncertainties correspond to block-bootstrap standard errors. For direction-averaged P and T rows, uncertainties include both the finite-sampling contribution and the dimer-to-dimer scatter, as described below.

For each method and transfer direction, reorganization energies were first computed separately for the two symmetry-related dimers. The reported direction-averaged value was then calculated as

$$\bar{\lambda}^j = \frac{1}{2}\left(\lambda_1^j + \lambda_2^j\right), \qquad j \in \{\mathrm{st}, \mathrm{var}\},$$

where λ_1^j and λ_2^j denote the values for the two symmetry-related dimers for a given direction.

Uncertainties were estimated as follows. For each individual dimer, finite-sampling uncertainties were obtained by block bootstrapping the corresponding $\Delta E_{M\to N}(t)$ trajectory. Each trajectory (total length 1 ns, positions saved every 4 fs) was divided into non-overlapping contiguous 20 ps blocks. For each bootstrap replica, blocks were resampled with replacement, using the same number of blocks as in the original trajectory, and λ^j was recomputed. The standard deviation of this bootstrap distribution gives the uncertainty reported for the individual-dimer rows in Table S1, where shown.

For the direction-averaged P and T values, blocks were resampled with replacement independently for the two dimers, λ^j was recomputed for each resampled dimer trajectory, and the two resulting values were averaged. The standard deviation of this bootstrap distribution was taken as the finite-sampling standard error of the

direction-averaged value, σ_{boot}.

Because the two dimers are symmetry-related but sampled by finite trajectories, we also included the residual dimer-to-dimer scatter in the uncertainty estimate. For two dimers, this contribution is

$$\sigma_{\text{dimer}} = \frac{|\lambda_1^j - \lambda_2^j|}{2}.$$

The final standard error reported for the direction-averaged P and T values was obtained by adding the two contributions in quadrature,

$$\sigma_{\text{tot}} = \sqrt{\sigma_{\text{boot}}^2 + \sigma_{\text{dimer}}^2}.$$

We note that for λ^{st}, which converges much faster than λ^{var}, all statistical uncertainties are well below 1 meV and are therefore not reported.

Figures S1 and S2 show the spectral density functions of vertical energy gap fluctuations and the cumulative reorganization energies for the individual P- and T-type dimers, respectively. These plots correspond to Fig. 2 of the main text, but are resolved into the individual dimers $P1/P2$ and $T1/T2$. The two symmetry-related dimers show very similar spectral features and cumulative reorganization energies in each case.

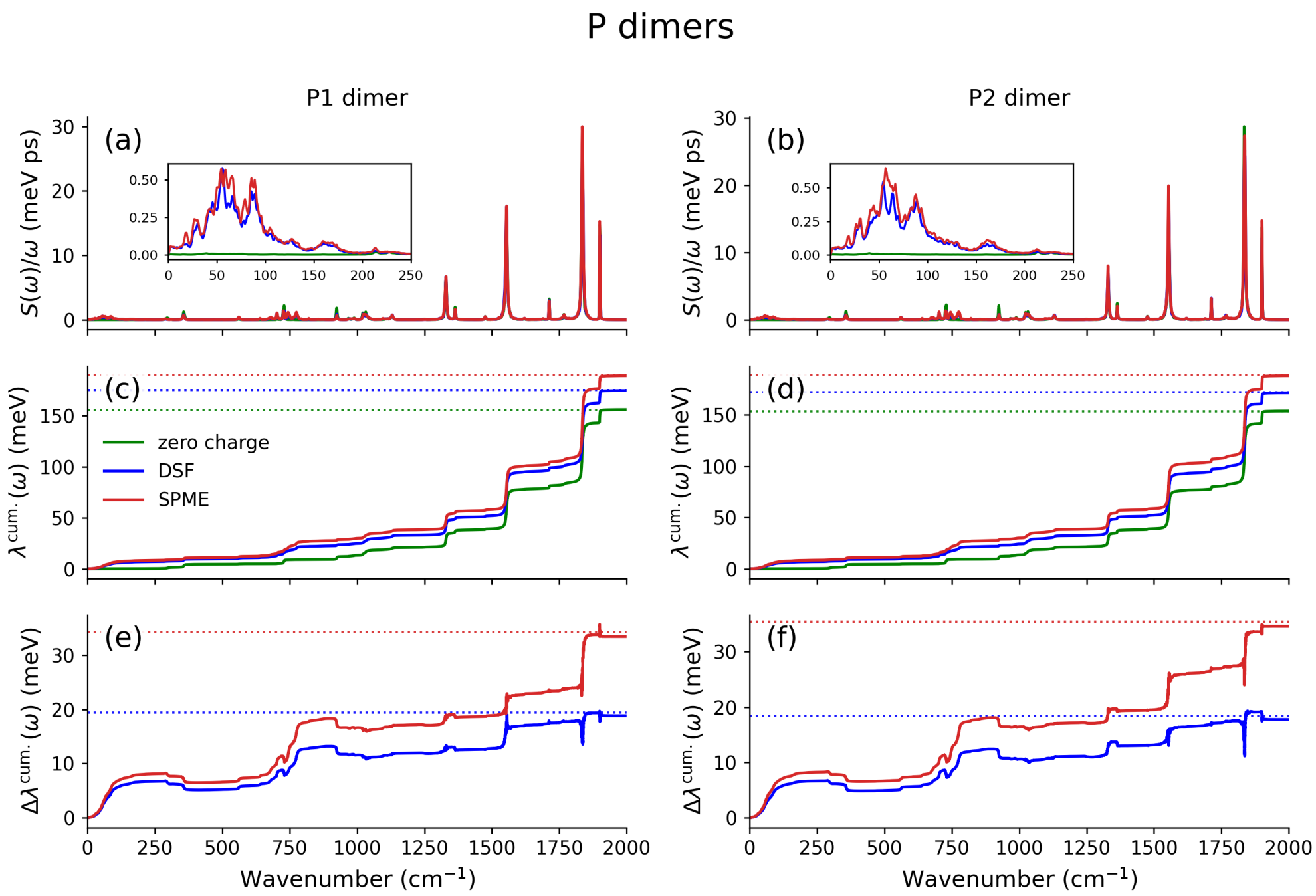

Figure S1 Spectral density functions of vertical energy gap fluctuations and cumulative reorganization energies for hole transfer using different treatments of Coulombic interactions for P-type dimers. (a,b) Spectral densities for transfer along the $P1$ and $P2$ directions, respectively. Insets highlight the low-frequency region from 0 to 250 cm^{-1}. (c,d) Cumulative fluctuation-derived reorganization energies up to frequency ω, $\lambda^{\text{cum}}(\omega)$, for transfer along the $P1$ and $P2$ directions, respectively. The dotted horizontal lines indicate the corresponding λ^{var} values obtained from the full energy-gap distribution. (e,f) Electrostatic contributions to the cumulative reorganization energy $\Delta\lambda^{\text{cum}}(\omega) = \lambda_j^{\text{cum}}(\omega) - \lambda_{\text{zero charge}}^{\text{cum}}(\omega)$ (j = DSF, SPME in blue and red, respectively), for transfer along the $P1$ and $P2$ directions, respectively. Dotted lines indicate the values of $\Delta\lambda^{\text{var}} = \lambda_j^{\text{var}} - \lambda_{\text{zero charge}}^{\text{var}}$ (j = DSF, SPME in blue and red, respectively).

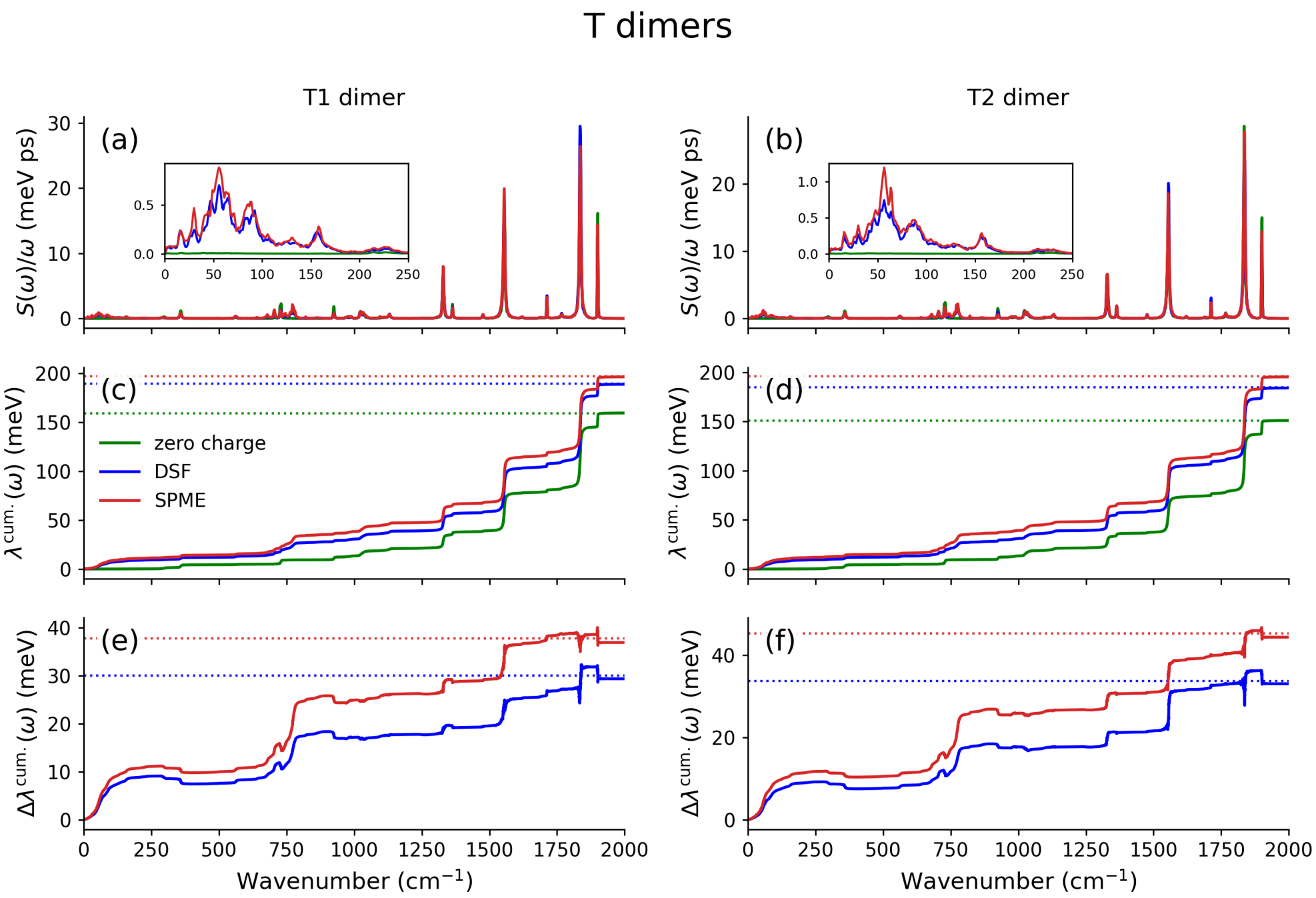

Figure S2 Spectral density functions of vertical energy gap fluctuations and cumulative reorganization energies for hole transfer using different treatments of Coulombic interactions for T-type dimers. (a,b) Spectral densities for transfer along the $T1$ and $T2$ directions, respectively. Insets highlight the low-frequency region from 0 to 250 cm^{-1}. (c,d) Cumulative fluctuation-derived reorganization energies up to frequency ω, $\lambda^{\text{cum}}(\omega)$, for transfer along the $T1$ and $T2$ directions, respectively. The dotted horizontal lines indicate the corresponding λ^{var} values obtained from the full energy-gap distribution. (e,f) Electrostatic contributions to the cumulative reorganization energy $\Delta\lambda^{\text{cum}}(\omega) = \lambda_j^{\text{cum}}(\omega) - \lambda_{\text{zero charge}}^{\text{cum}}(\omega)$ (j = DSF, SPME in blue and red, respectively), for transfer along the $T1$ and $T2$ directions, respectively. Dotted lines indicate the values of $\Delta\lambda^{\text{var}} = \lambda_j^{\text{var}} - \lambda_{\text{zero charge}}^{\text{var}}$ (j = DSF, SPME in blue and red, respectively).
